# Targeting Dendritic Cell Function during Systemic Autoimmunity to Restore Tolerance

**DOI:** 10.3390/ijms150916381

**Published:** 2014-09-16

**Authors:** Juan P. Mackern-Oberti, Fabián Vega, Carolina Llanos, Susan M. Bueno, Alexis M. Kalergis

**Affiliations:** 1 Millennium Institute of Immunology and Immunotherapy, Departamento de Genética Molecular y Microbiología, Facultad de Ciencias Biológicas, Pontificia Universidad Católica de Chile, Portugal 49, Santiago 8330025, Chile; E-Mails: jmackern@bio.puc.cl (J.P.M.-O.); sbueno@bio.puc.cl (S.M.B.); 2 Departamento de Inmunología Clínica y Reumatología, Escuela de Medicina, Pontificia Universidad Católica de Chile, Marcoleta 350, Santiago 8330033, Chile; E-Mails: fab.vega.t@gmail.com (F.V.); cllanosm@gmail.com (C.L.); 3 INSERM, UMR 1064, Nantes 44093, France

**Keywords:** autoimmunity, tolerance, therapy, dendritic cells

## Abstract

Systemic autoimmune diseases can damage nearly every tissue or cell type of the body. Although a great deal of progress has been made in understanding the pathogenesis of autoimmune diseases, current therapies have not been improved, remain unspecific and are associated with significant side effects. Because dendritic cells (DCs) play a major role in promoting immune tolerance against self-antigens (self-Ags), current efforts are focusing at generating new therapies based on the transfer of tolerogenic DCs (tolDCs) during autoimmunity. However, the feasibility of this approach during systemic autoimmunity has yet to be evaluated. TolDCs may ameliorate autoimmunity mainly by restoring T cell tolerance and, thus, indirectly modulating autoantibody development. *In vitro* induction of tolDCs loaded with immunodominant self-Ags and subsequent cell transfer to patients would be a specific new therapy that will avoid systemic immunosuppression. Herein, we review recent approaches evaluating the potential of tolDCs for the treatment of systemic autoimmune disorders.

## 1. Introduction

Central and peripheral immune tolerance are key mechanisms responsible for avoiding the initiation of immune responses against self-antigens [1]. Although much progress has been made in understanding the immunological pathways underlying autoimmunity, current therapies for systemic autoimmune diseases have not been improved [2,3]. Although it is widely known that dendritic cells (DCs) play a crucial role at initiating the immune response against pathogens, this cell type also contributes to maintain peripheral immune tolerance [4]. Chronic progression and complexity of systemic autoimmune diseases, such as Systemic Lupus Erythematosus (SLE) and Rheumatoid Arthritis (RA) has dampened the development of new specific therapies. SLE preferentially affects women and is characterized by the presence of a wide spectrum of symptoms, including vasculitis, glomerulonephritis, serositis, skin lesions and central nervous system involvement. It is known that many immune cell types contribute to SLE pathogenesis [5,6,7,8]. Our group, as well as others, has reported that DCs from SLE patients show increased expression of co-stimulatory molecules, as well as a higher ratios of activating to inhibitory Fc gamma receptors (FcγRs) as compared to healthy controls. These data suggest that DCs may be involved in the initiation of SLE pathogenesis [6,9,10].

Experimental therapies for SLE, based on monoclonal antibodies, have failed to show the promising results observed for other autoimmune diseases, such as rheumatoid arthritis (RA), anti-neutrophil cytoplasm antibody (ANCA)-associated vasculitis and type 1 diabetes [11,12]. For instance, the latest results of phase III clinical trials of the new biological agent belimumab, a monoclonal antibody that blocks the soluble B-lymphocyte stimulator (BLyS), has shown positive effects lasting through 52 weeks, nevertheless, benefits from treatment did not result in improvement when compared to placebo at week 76. Furthermore, belimumab produces deletion of naïve B and plasma cells, but not memory cells, which is likely to impair anti-microbial immunity and render the patient susceptible to infections [13,14,15,16].

The use of DCs for immunotherapy has become an attractive possibility for the treatment of autoimmune diseases in an Ag-specific manner, which is thought to avoid both systemic immunosupression and the adverse effects of steroids [17,18,19]. In this review, we discuss current approaches relative to the use of *in vitro* generated tolerogenic DCs (tolDCs) as a therapeutic approach for systemic autoimmune diseases.

## 2. Targeting DC-T Cell Interactions to Prevent Autoimmunity

**Figure 1:**
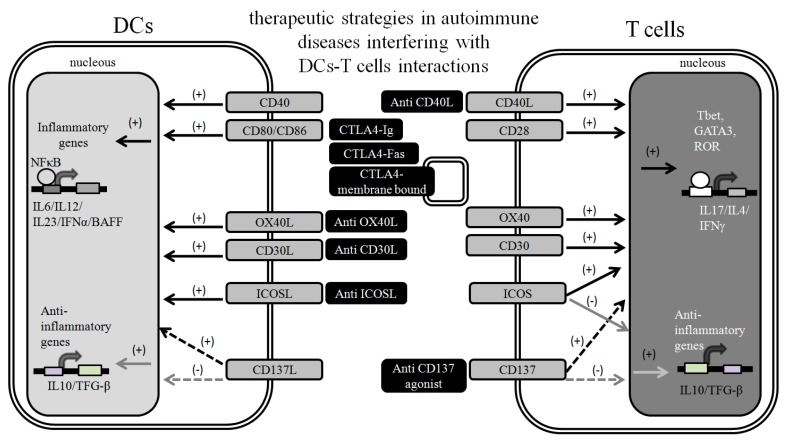
Modulation of DC-T cell interactions as a therapeutic strategy. T cells, key effectors of immunity, depend on signals on the surface of DCs and other APCs to become activated. The process of T cell activation may be modulated to prevent the exacerbated inflammatory activity in autoimmune diseases and restore tolerance. This goal can be achieved by blockage of activating molecules and receptors on DCs or T cells resulting in decreased expression of inflammatory genes and transcription factors involved in effector T cell commitment, while inducing expression of anti-inflammatory genes. Cross-linking inhibitory receptors with antibodies or ligands is another interesting way to reduce T cell activity. A complete understanding of the function of co-stimulatory and co-inhibitory molecules and respective receptors and their role in autoimmune pathogenesis will help to establish more efficient approaches for immunotherapy. Black arrows indicate inflammatory pathways. Grey arrows indicate anti-inflammatory pathways.

In autoimmune susceptible individuals, the autoreactive immune response is possibly initiated when antigen presenting cells (APCs) present self-Ags to autoreactive T cells that have leaked from thymic central and peripheral tolerance [1,20]. APCs, including DCs, express crucial molecules for T cell priming, such as peptide-MHC complexes and the co-stimulatory molecules CD40, CD80, and CD86. Activated CD4^+^ T cells interact with Ag-specific B cells and promote the initiation of the humoral response [21,22,23,24,25]. CD80/CD86 binding to CD28 expressed on T cells leads to full activation, IL-2 production and cell proliferation [26,27]. Interestingly, DCs from lupus patients show higher expression of co-stimulatory molecules, such as CD86 and CD40, than DCs from healthy controls suggesting an immunogenic prone state for these cells [6,28]. Furthermore, blockade of ligand-receptor interactions at the APC-T cell interface, including OX40-OX40L and CD30-CD30L engagement, can lead to a delay of autoimmune disease onset by inhibiting the expression of pro-inflammatory cytokines, such as IFN-γ and IL-4 and a subsequent reduced leukocyte infiltration into peripheral tissues [29,30]. Furthermore, it has been reported that targeting CD40-CD40L interactions between APCs and T cells by the administration of an anti-CD40L mAb can significantly ameliorate symptoms of autoimmune diseases including Experimental Autoimmune Encephalitis (EAE) and uveo-retinitis [31,32]. In addition, *in vitro* blockade of ICOS/ICOS-L interaction inhibits IL-10 release by T cells without affecting IL-2 production [33]. ICOS/ICOS-L ligation modulates T cell proliferation, survival and polarization [34,35]. In contrast, regulatory T cells (Treg) may also express ICOS, indicating that the ICOS/ICOS-L axis can influence effector T cell responses [36]. Interestingly, it has been shown that *ICOS*^−/−^ and *ICOS-L*^−/−^ NOD mice were protected from spontaneous diabetes [37]. However these mice strains developed other autoimmune symptoms related to neuromuscular disorders, suggesting that ICOS/ICOS-L signaling would play a crucial role in regulating immune tolerance by modulating the balance between Treg cells and diabetogenic effector T cells (Figure 1).

CTLA-4 engagement with CD80/CD86 negatively regulates TCR signaling and T cell function and promotes immune tolerance. Therefore, modulating this molecular interaction could be a powerful regulator of the immune response. This notion is supported by the phenotype shown by CTLA-4 deficient mice, which develop massive lymphoproliferation and autoimmunity [38]. Major progress has been made in designing new therapies for autoimmune diseases with the use of CTLA-4-related biological agents [39]. Immunosuppressive effects of CTLA-4 settle in the blockage of CD28-CD80/CD86 interaction by binding to CD80/CD86 on the DCs. Different forms of CTLA-4 have been designed, such as CTLA-4-Ig, CTLA-4-Fas and membrane-bound anti-CTLA-4 antibody [39,40]. CTLA-4-Ig (Abatacept) treatment is used in RA and has been extensively evaluated in different autoimmune disorders and new clinical trials are being conducted in T1D patients [12,39,41]. An other interesting fusion protein is CTLA-4-FasL which may bind to CD80/CD86 on APCs triggering activation-induced cell death on activated T cells by Fas ligation [42,43]. Interestingly, the administration of adenovirus vectors expressing CTLA-4-FasL ameliorated pancreatic insulitis, by inducing apoptosis of pancreatic T cells and ameliorating the immune response against pancreatic antigens [44]. Additionally, it has been reported that the expression of membrane-bound anti-CTLA-4 antibody on B cells in NOD mice prevented the development of spontaneous autoimmune diabetes [40] (Figure 1).

Programmed cell death-1 (PD-1) molecule is another inhibitory receptor expressed by T cells and binds to PD-L1 and PD-L2, which are expressed on DCs and other APCs [45]. It is known that DCs can inhibit T cell activation by PD-L1-PD-1 interaction, as well as promoting Treg cell development [46,47,48]. Furthermore, it has been reported that immature DCs prevent experimental autoimmune encephalomielitis (EAE) by the induction of PD-1^+^Tregs cells [49,50]. PD-L1 deficiency enhances IFN-γ production by CD4^+^ T cells and the activation of CD8^+^ T cell responses, conferring an increased susceptibility to autoimmunity [51]. In addition, mice lacking PD-1 develop autoimmune symptoms as those observed during SLE, including glomerulonephritis and lymphoproliferative disorders [52].

B and T lymphocyte attenuator (BTLA) is an inhibitory receptor that modulates lymphocyte activation [53]. Mice lacking this receptor show leukocyte infiltration of several tissues similarly as observed in Sjögren’s syndrome and autoimmune hepatitis [54]. Toso/Faim3 is a surface molecule expressed on lymphocytes and myeloid cells that has been implicated in the regulation of Fas- and TNF receptor (TNFR)-dependent T cell apoptosis [55]. Interestingly, Toso deficient mice shows a decreased susceptibility to develop EAE, due to lower CD4^+^ and CD8^+^ T cell responses, suggesting that Toso is a crucial mediator of inflammatory autoimmune responses [56]. Although CD137-CD137L ligation between T cells and APCs leads to cellular activation, CD137 deficiency in MRL/lpr lupus murine model paradoxically induces an accelerated disease [57]. Furthermore, *in vivo* administration of agonistic anti-CD137 monoclonal antibody to lupus mice reduces symptoms, strongly suggesting that CD137-CD137L is involved in immune regulation and tolerance [58] (Figure 1).

On the other hand, it has been shown that APCs expressing CD2 without surface co-stimulatory molecules could promote the differentiation of Tregs, which produce high amounts of IL-10 and suppresses T cell responses [59]. In contrast, it has been reported that IL-6 produced by DCs play a critical role in the activation of effector T cell, as well as limiting Treg-mediated suppression [60,61]. The molecular mechanism underlying Treg modulation by DCs is unknown but it is thought that is independent of co-stimulatory molecules [60]. In the Sle1/Sle2/Sle3 lupus murine model, lymphoid tissues show higher numbers of DCs producing IL-6, which may promote effector T cell priming while impairing Treg cell function [61].

It has been reported that DCs play a crucial role in T cell priming during lupus development. Interestingly, the transfer of DCs loaded with apoptotic antigens could initiate a transient autoreactive immune response in autoimmune resistant mice and systemic autoimmunity in susceptible strains [62,63,64]. Understanding the complex scenario of activation and inhibitory molecules simultaneously expressed on DCs is crucial to design new therapies for autoimmune diseases based in autologous DCs transfer.

## 3. Targeting DC-B Cell Interactions to Prevent Autoimmunity

**Figure 2:**
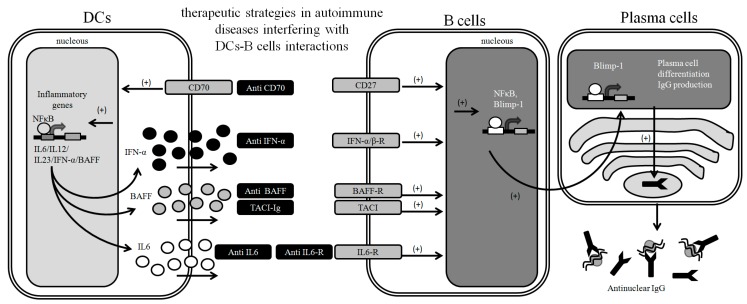
Modulation of DC-B cell interactions as a therapeutic strategy. Interactions between DCs and B cells are poorly understood yet, but increasing number of reports remark the relevance of DC-B cell communication in the onset of SLE and other autoimmune diseases. Engage of CD27 by CD70 expressed on pDCs induces B cell differentiation into plasma cells, which secrete high amounts of immunoglobulin. Additionally, DCs secrete soluble cytokines, which trigger B cell activation, proliferation and differentiation into plasma cells. Obstruction of these signals may prove to be beneficial as therapy for autoimmune diseases in which autoantibodies production is involved such as SLE.

Although T-B cells interactions has been extensively studied, much less data on DCs-B cells crosstalk is known. One of the most important findings of B cell biology is the discovery of the B‑cell survival and maturation factor, B cell-activating factor of the TNF family (BAFF) (also known as B-lymphocyte stimulator (BLyS)) and the development of BAFF-blocking monoclonal antibody (belimumab) in clinical practice for lupus disease treatment [15,65]. Lupus patients with nephritis and central nervous system affections show higher levels of BAFF than lupus patients with other organ involvement suggesting an active role in autoimmune pathogenesis [66]. Similarly, patients with myasthenia gravis, Grave’s disease, anti-GBM syndrome and anti-neutrophil cytoplasmic autoantibody associated vasculitis show increased serum levels of BAFF [67,68,69,70]. While BAFF deficiency in mice leads to immunodeficiency, BAFF overproduction leads to an increase in mature B cells, and auto-antibodies, subsequently triggering a lupus-like disease [65,71]. In addition, the administration of TACI-Ig (a soluble form of BAFF receptor) in a lupus murine model prevents glomerulonephritis and prolongs survival of lupus mice [72]. However a clinical trial based in the administration of TACI-Ig (atacicept) in patients with active lupus nephritis had to be stopped due to infectious disease onset secondarily to IgG depletion [73] (Figure 2).

Interestingly, DCs (among other immune cells) can produce high amounts of BAFF and APRIL, suggesting that the B cell response could be modulated by innate immune cells [74]. It has been reported that DCs activated with IFN-α and IL-6 may produce IL-12, IL-6 and BAFF that in turn induces B cell differentiation to plasma cell and Igs production [75]. In addition, the administration of tocilizumab, a humanized antibody that blocks IL-6 function by targeting the IL-6 receptor, ameliorate clinical outcome in RA and systemic juvenile idiopathic arthritis [76,77]. In RA patients, the administration of tocilizumab ameliorates synovitis leading to a reduction in joint damage. [78]. Additionally, many anti-IL-6 or anti-IL-6-R have been developed and tested for immunosupression of autoimmune diseases [79]. Moreover, human DCs and monocytes induce BAFF production after IFN-α and IFN-γ stimulation while monocytes from lupus patients show higher production of BAFF than healthy controls [80,81]. Furthermore, anti-IFN-α therapy in lupus patients decreased BAFF mRNA levels [82]. Also, it is known that BAFF levels are higher in CD11c^+^ cells from female than male mice and estrogen stimulation of immune cells induced BAFF mRNA and protein levels, thus linking BAFF with the sex female bias [83]. Most importantly, DCs could transfer Ags and ICs to naive B cells in lymphoid organs in order to initiate the humoral immune response [84]. Moreover, it has been reported that DCs may induce IgA class switch after CD40 ligation of naive B cells, suggesting that DCs directly modulate B-T cell cooperation [85]. All these data on lupus patients and murine models strongly suggest an association between BAFF, IFN-α and DCs (Figure 2).

Similarly to IFN-α, DCs stimulated with TLR4 ligand induce B cell proliferation, antibody production and chemokine receptors expression which are involved in cell trafficking to germinal centers leading to IgG production [86]. In addition, it has been shown that the interaction of CpG-stimulated pDCs with B cells induced the expression of co-stimulatory molecule CD86, thus suggesting an active role in the modulation of T cell priming [87]. On the other hand, B cells could also modulate DCs function. It has been reported that activated B cells could modulate the expression of MHCII, CD80, CD86 and the production of IL-12p70 on DCs, which in turn prevents DC-induced T-cell proliferation [88].

It has been reported that CD27-CD70 interaction is crucial for B cell-DC crosstalk. It has been reported that CpG stimulation of pDCs induces IFN-α and CD70 expression, which in turn leads to plasma cell differentiation and antibody production on B cells [89]. Interestingly, when B cell-DCs interaction CD27-CD70 was antagonized by the administration of an anti-CD70 antibody, the ability of activated pDCs to induce B cell proliferation was significantly reduced, which is highly relevant for the design of new immunosuppressive therapies [89]. Studies in mice have shown that CD70 ligation with anti-CD70 antibody induces B cell proliferation and that the administration of anti-CD70 or the presence of its ligand CD27 induce substantial B cell activation [90] (Figure 2).

The role of DCs in autoimmune development and autoantibody production has been highlighted by studies using a CD11c-specific diphtheria toxin-α chain system to delete DCs [91]. It has been currently shown that DCs depletion ameliorates lupus disease including kidney infiltration and decreases renal damage in the MRL. Fas*^lpr^* lupus murine model [91]. Surprisingly, DCs are crucial for plasmablast generation and Ig class switching [91]. In the other hand, a subset of splenic tolerogenic DCs may induce regulatory B cell differentiation that produces high amounts of IL-10 and could regulate T cell responses [92].

Taken together, these data highlight the potential of designing new therapies targeting DCs as well as protocols based in the generation of DCs expressing a tolerogenic phenotype that could modulate B cells, plasma cell differentiation and Ig production, which is essential for an efficient therapy against systemic autoimmunity.

## 4. DC Abnormalities in Human Autoimmune Diseases

Plasmacytoid DCs (pDCs) and conventional DCs (cDCs) have been reported to show abnormalities in patients with autoimmune diseases. [93,94]. During multiple sclerosis (MS), cDCs showed an increased expression of co-stimulatory molecules such as CD80 and CD40; an increase secretion of pro-inflammatory cytokines such as IL-12 and TNF-α; and a decreased expression of PD-L1, suggesting an active role in T cell priming [94].

Lupus patients show lower numbers of blood cDCs as compared to healthy control while pDCs are increased, suggesting that this cell type is affected [95,96,97]. Our group has shown lupus patients have an increased expression of co-stimulatory molecules such as CD40 and CD86, suggesting that DCs immunogenicity is augmented [6]. cDCs from lupus patients show higher expression of activating FcγRs and lower expression of the inhibitory FcγRIIb, which correlates with the activity index of SLE (SLEDAI) [6]. Several studies on lupus patients report that DCs show an aberrant phenotype, mostly dominated by a high expression of co-stimulatory molecules [9,10]. In addition, the expression of inhibitory receptors have been reported to be decreased in DCs from lupus patients such as the expression of LAIR-1 on pDCs of juvenile lupus patients [98]. Contrary to expected, it has been recently reported that Mer, a receptor involved in the process of apoptotic cell recognition and removal, is increased in DCs from lupus patients and corticosteroids may induce Mer expression, favoring its beneficial effects in SLE [99]. Synovial fluid from active RA patients showed a subpopulation of DCs that expressed an activated phenotype with high expression of co-stimulatory molecules such as CD80, CD83 and CD86 [100].

During type 1 diabetes onset, it has been reported that the number of both cDCs and pDCs were decreased in peripheral blood, showing a altered chemokine receptor expression [101].

Poly:IC and CpG ligation of TLR7 and TLR9 on pDCs endosomes produces high amounts of type I IFN via IRF7 signaling pathway [102,103,104]. Salivary glands from Sjögren’s Syndrome (SS) patients show higher levels of IFN-inducible genes, such as TLR8, TLR9, IFITM1, BAFF and BCMA, which may be crucial for DCs maturation, B cell activation and the subsequent T cell priming [105]. Furthermore, patients with SS showed an increase infiltration of pDCs in salivary glands highlighting a major role for this cell type during autoimmunity [105]. The role of IFN-α in triggering autoreactive immune responses or lupus-like syndrome has been linked to the clinical finding that patients receiving IFN-α therapy for non-autoimmune diseases may develop anti-nuclear antibodies and glomerulonephritis [106,107,108,109].

Understanding the complex network of co-stimulatory/co-inhibitory receptors and cytokine signaling on DCs and T cells that leads to the activation or regulation of the immune response will favor the designing of new therapeutic targets for autoimmune disease treatments.

## 5. DC Maturation Stimuli during Autoimmunity

Toll like receptors (TLRs) may recognize pathogen associated molecular patterns (PAMPs) as well as endogenous molecules released during stress conditions and cell death such as HMGB1, HSP60-70, fibronectin, fibrinogen, hyaluronic acid fragments, ssRNA and immunocomplexes (ICs) containing chromatin [110,111,112]. TLR engagement may lead to the production of proinflammatory factors leading to tissue damage. Interestingly, during lupus pathogenesis, circulating immunocomplexes (IC) may be recognized by DCs and other immune cells promoting inflammation and tissue injury in SLE. ICs containing HMGB1 are crucial for anti-dsDNA development in SLE by a mechanism likely to be driven by a TLR2/MyD88 dependent pathway [111]. It is proposed that self-DNA/self-RNA from dying cells can be internalized and transported into TLR7 or TLR9 containing endosomes in DCs leading to IFN-α production and initiating the autoreactive immune response [113,114,115,116]. Self-DNA may form macromolecular aggregates reaching TLR9 expressing endosomes and break immune tolerance to self-DNA leading to the onset of autoimmune disorders such as psoriasis, arthritis and SLE [117,118]. Hydroxychloroquine (HCQ), a drug currently used in lupus treatment, increases cytoplasmic pH preventing acidification and maturation of endosomes while decreases pro-inflammatory cytokines production upon TLR7 and TLR9 ligation in DCs [119,120]. Probably, HCQ inhibits IFN-α production by limiting endosome maturation and the binding of TLRs to ICs containing self-DNA/RNA [121]. An unbalanced activating/inhibitory FcγR signaling in SLE patients may significantly influence DC immunogenicity due to the presence of ICs containing apoptotic cells and the deficiency in the clearance apoptotic bodies [8]. It has been shown that TLRs expressed by pDCs recognize RNA and DNA when added along with serum IgGs from lupus patients while serum from healthy controls do not induced DCs activation suggesting that ICs, FcγRs and TLRs play a crucial role during lupus pathogenesis [121,122,123]. Recently, it has been reported that different molecules could bind to self antigens present in circulating ICs, such as C-reactive protein (CRP) and the antimicrobial peptide LL-37, a cathelicidin polypeptide being capable of inhibiting or promoting the IFN-α response, and mediate endosomal TLR recognition of ICs [115,116,124]. DCs maturation by TLR agonists can have a negative impact in peripheral tolerance especially in regulatory T cell (Treg) function. It has been reported that Treg function could be abolished by pDCs activated by CpG and simultaneously driving to Th17 cells expansion thereby promoting a pro-inflammatory response [125,126,127,128].

## 6. Designing New Therapies Based on Tolerogenic DCs

Although a great deal of progress has been made in experimental approaches using tolerogenic DCs to ameliorate tissue specific autoimmune diseases, the efficacy of tolDCs at suppressing systemic autoimmunity still remains to be assessed. Different strategies have been used promote immunosuppression by DCs-based therapy such as T helper bias, Treg differentiation and T cell anergy [129,130,131]. One of the most interesting features of tolDCs based therapy is the potential of loading DCs with immunodominant self-antigens responsible for autoimmune mediated damage avoiding systemic immunosuppression, such as observed under corticoids treatment [132].

Co-stimulatory molecule expression and cytokine production are crucial for T cell immunosuppression by tolDCs and currently it is known that tolDCs phenotype will be characterized by a low expression of MHC-II, CD40, CD80, CD86; a concomitant reduced production of pro-inflammatory cytokines IL-6 and IL-12; and increased secretion of the anti-inflammatory cytokine IL-10 [129,132,133].

Murine DCs are most frequently generated in *in vitro* cultures from bone marrow precursors with GM-CSF [134]. In human, DCs are generated *in vitro* from blood CD14^+^ monocytes cultures with recombinant GM-CSF and IL-4 [135,136]. After DCs differentiation it is possible to generate tolDCs *in vitro* by several methods, such as metabolic control, pharmacologic intervention, biological agents, and gene therapy [137,138]. Interestingly, new approaches are being conducted to design microparticulate systems for specific delivery of tolerogenic agents to DCs. A delivery of multiple tolerogenic factors can be performed by polylactic-co-glycolic acid microparticles of different phagocytosable and unphagocytosable microparticles [139].

### 6.1. Metabolic Control

**Figure 3:**
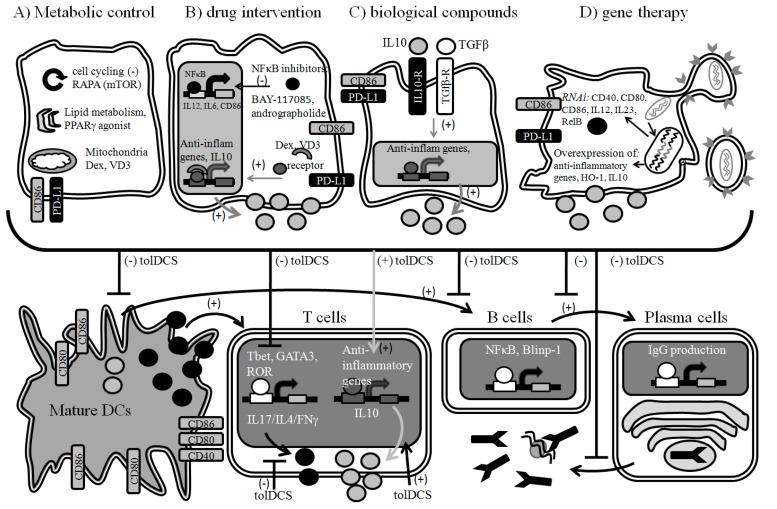
Current strategies to generate tolDCs. (**A**) Metabolic control of different cellular processes such as inhibition of mitochondiral phosphorylation (Dexamethasone- Dex), glycolysis, lipid metabolism (PPARγ agonists such as rosiglitazone) and cell cycling (rapamycin); (**B**) Drug intervention to promote the induction of tolDCs is mainly achieved by drugs, which interfere with NF-κB signaling pathway (Dex-dexamethasone, VD3-vitamin D3, aspirin, BAY11-7082). NF-κB signaling pathway is a crucial event during DC activation and maturation process; (**C**) Biological compounds such as the anti-inflammatory cytokines IL-10 and TGF-β are powerful tolerogenic agents which induce tolDCs with the capacity of secrete high levels of anti-inflammatory cytokines; (**D**) The modification of DCs with RNAi and lentivirus (or adenovirus) vectors offers new approaches to generate tolDCs. By the transduction of RNAi specific for pro-inflammatory cytokines (IL12, IL23) or co-stimulatory molecules (CD40 and CD86), the immunogenicity of DCs is severely affected. In contrast, the transduction of lentiviral (or adenoviral) vectors containing anti-inflammatory genes, such as heme oxygenase-1(HO-1) or IL-10, could also induce the generation of tolDCs which keep the capacity of produce anti-inflammatory cytokines with a low expression of co-stimulatory molecules. Generally, independent of the protocol used to induce the tolerogenic phenotype, tolDCs are resistant to pro-inflammatory stimuli. Remarkably, when tolDCs interact with T cells, they prevent cellular activation, proliferation and the production of pro-inflammatory cytokines such as IL-4, IL-17 and IFNγ while inducing (or no effect on) the production of IL-10. In addition, tolDCs could also interact with B cells reducing activation, plasma cell differentiation and the production of immunoglobulins. All these data promote tolDCs as a potential approach for the treatment of systemic autoimmune diseases in which both T and B cells responses are deregulated. Black arrows indicate inflammatory pathways. Grey arrows indicate anti-inflammatory pathways. Blunted lines indicate inhibition.

It is known that changes in cellular activation may initiate different intracellular processes leading to changes in global metabolism and targeting these mechanism of cellular metabolism can be exploited to shape a desired immune responses or immunosuppression [138]. Thus, in theory the inhibition of early metabolic process during DCs maturation will reset DCs activation, migration, and T cell priming. After TLR ligand stimulation, DCs undergo a metabolic switch with an increase in glycolysis and a concomitant progressive loss of mitochondrial oxidative phosphorylation [140]. Interestingly, the administration of dexamethasone and vitamin-D3 to DCs induces the expression of genes associated with mitochondrial metabolism and oxidative phosphorylation [141] (Figure 3A). Although not much evidence exists about the efficiency of generating tolDCs through interfering with metabolic pathways the most studied molecular targets are mTOR, HIF-1a, AMPK and PGC1 [138,142,143,144,145] (Table 1). Additionally, rosiglitazone, a PPARγ agonist, which is known to display a tolerogenic capacity on DCs such as the amelioration of EAE, may regulate their function by altering lipid metabolism [146,147,148,149] (Figure 3A). Targeting metabolic pathways in DCs could be also implemented in the design of tolDCs-based immunotherapies (Table 1).

**Table 1:** Experimental strategies for the induction of tolDCs in autoimmune diseases. CIA: collagen-induced arthritis; EAE: experimental autoimmune encephalomyelitis; IL: interleukin.

Agent	Protocol				Type of Tolerogenic Response	Targeted Disease	Reference
	Species	Differentiation	Relevant Antigen	Type of Study			
Dexamethasone and Vitamin D3	human	Blood monocytes, GM-CSF and IL-4, 5–6 days	alloantigen	*in vitro*; pre-clinical	Maturation-resistant phenotype, IL10/IL12; Impact in metabolism (lipids, glucose and oxidative phosphorylation); Migratory phenotype alterations; Reduce T cell priming and allospecific T cell response	Immune-mediated diseases; Prevention of graft rejection; Rheumatoid arthitis; Sjogren syndrome	Ferreira *et al.*, 2011 [141]; Volchenkov *et al.*, 2013 [148]; Volchenkov *et al*., 2013 [150]; Xing *et al*., 2002 [151]; Unger *et al*., 2009 [152]; García-González *et al*., 2013 [153]
	mouse	Bone marrow, GM-CSF, 5 days	-	*in vivo*	T cell priming; Maturation-resistant phenotype, IL10/IL12; Reduction of proinflammatory chemokines and cytokines	Immune-mediated diseases	Xing *et al*., 2002 [151]
	mouse	Bone marrow, GM-CSF, 5 days	-	*in vitro*	T cell priming; Maturation-resistant phenotype, IL10/IL12	Immune-mediated diseases	Moser *et al*., 1995 [154]
Dexamethasone plus monophosphoryl lipid A	human	Blood monocytes, GM-CSF and IL-4, 5–6 days	alloantigen	*in vitro*; pre-clinical	Stable phenotype and migratory capacity to lymphoid chemokines; T cell priming; Maturation-resistant phenotype, IL10/IL12	Rheumatoid arthitis; Immune-mediated diseases; Prevention of graft rejection	García-González *et al*., 2013 [153]
Dexamethasone	human	Blood monocytes, GM-CSF and IL-4, 5–6 days	-	*in vitro*	Maturation-resistant phenotype, IL10/IL12; T cell priming	Immune-mediated diseases	Rea *et al*., 2000 [155]
Vitamin D3	mouse	Bone marrow, GM-CSF, 5 days	-	*in vivo*	Reduce EAE severity; Maturation-resistant phenotype, IL10/IL12; Regulatory T cell induction	EAE; Autoimmunity	Farías *et al*., 2013 [156]; Unger *et al*., 2009 [152]
	human	Blood monocytes, GM-CSF and IL-4, 5–6 days	myelin peptides	*in vitro*	Maturation-resistant phenotype, IL10/IL12; Reduce autoreactive T cell induction	MS; Autoimmunity	Raïch-Regué *et al*., 2012 [157]
Rapamycin	mouse	Bone marrow, GM-CSF, 5 days	alloantigen	*in vitro*	Maturation-resistant phenotype; Reduce T cell priming and allospecific T cell response	prevention of graft rejection	Turnquist *et al.*, 2007 [143]; Taner *et al.*, 2005 [144]; Hackstein *et al.*, 2003 [158]		
	mouse	Bone marrow, GM-CSF, 5 days	alloantigen	*in vivo*	Reduce survival of alloantigen-specific CD8+ T cells *in vivo*	Prevention of graft rejection	Fischer *et al.*, 2011 [145]		
	human	Blood monocytes, GM-CSF and IL-4, 5–6 days	alloantigen	*in vitro*	Maturation-resistant phenotype; Reduce T cell priming and allospecific T cell response	Immune-mediated diseases	Fedoric *et al.*, 2008 [159]		
Andrographolide	mouse	Bone marrow, GM-CSF, 5 days	MOG peptide	*in vitro*	Reduce T cell priming and antigen processing; NF-κB inhibition	Autoimmunity; EAE	Iruretagoyena *et al.*, 2005 [146]		
	mouse	Bone marrow, GM-CSF, 5 days	MOG peptide	*in vivo*	Reduce EAE severity; NF-κB inhibition	Autoimmunity; EAE	Iruretagoyena *et al.*, 2006 [149]		
Aspirin	mouse	Bone marrow, GM-CSF, 5 days	alloantigen	*in vitro*	Maturation-resistant phenotype; IL10/IL12; Phagocytosis inhibition; Reduce T cell primi	Immune-mediated diseases	Hackstein *et al.*, 2001 [160]; Buckland *et al.*, 2006 [161]; Cai *et al.*, 2011 [162]		
Rosiglitazone	mouse	Bone marrow, GM-CSF, 5 days	MOG peptide	*in vivo*	Reduce T cell priming; Reduce EAE severity, NF-κB inhibition	Autoimmunity; EAE	Iruretagoyena *et al.*, 2006 [149]		
	human	Blood monocytes, GM-CSF and IL-4, 5–6 days	-	*in vitro*	Reduce proinflammatory cytokine expression; Lipid accumulation appears to be diminished in these cells	Immune-mediated diseases	Szatmari *et al.*, 2007 [147]		
Troglitazone	human	Blood monocytes, GM-CSF and IL-4, 5–6 days	-	*in vitro*	Maturation-resistant phenotype, IL10/IL12	Immune-mediated diseases	Volchenkov *et al.*, 2013 [148]		
Cobalt Protoporphyrin	human	Blood monocytes, GM-CSF and IL-4, 5–6 days	alloantigen	*in vitro*	Reduce T cell priming; Maturation-resistant phenotype, IL10/IL12; Reduce allospecific T cell response	Immune-mediated diseases; Prevention of graft rejection	Chauveau *et al.*, 2005 [163]
Bay 11-7082	mouse	Bone marrow, GM-CSF and IL-4, 5 days	methylated serum albumin	*in vivo*	Reduce disease severity; Reduce T cell response; NF-κB inhibition	CIA (Rheumatoid arthitis)	Martin *et al.*, 2007 [164]
	mouse	Bone marrow, GM-CSF, 5 days	-	*in vitro*	Maturation-resistant phenotype, IL10/IL12	Immune-mediated diseases	Ade *et al.*, 2007 [165]
Tacrolimus	mouse	Bone marrow, GM-CSF, 5 days	-	*in vivo*	-	CIA (Rheumatoid arthitis)	Ren *et al.*, 2014 [166]
	human	Blood monocytes, GM-CSF and IL-4, 5–6 days	-	*in vitro*	Maturation-resistant phenotype, IL10/IL12; Anti-inflammatory cytokine gene expression	Rheumatoid arthitis	Ren *et al.*, 2014 [166]
IL-10	human	Blood monocytes, GM-CSF and IL-4, 5–6 days	alloantigen; allergen	*in vitro*; pre-clinical	Maturation-resistant phenotype, IL10/IL12; Reduce T cell priming and allospecific T cell response	Systemic Lupus Erythematosus; Type 1 Diabetes; Immune-mediated diseases; Asthma and allergy	Sato *et al.*, 1999 [167]; Knodler *et al.*, 2008 [168]; Velten *et al.*, 2004 [169]; Kubsch *et al.*, 2003 [170]; Steinbrink *et al.*, 2002 [171]; Li *et al.*, 2010 [172]; Lopez *et al.*, 2011 [173]; Crispin *et al.*, 2012 [28]
	mouse	Bone marrow, GM-CSF, 5 days	-	*in vitro*	Maturation-resistant phenotype	Immune-mediated diseases	Ruffner *et al.*, 2009 [174]
	rat	Bone marrow, GM-CSF, 5 days	-	*in vivo*	Maturation-resistant phenotype; Reduce T cell priming and allospecific T cell response	Prevention of graft rejection	Jiang *et al.*, 2004 [175]
TGF-β	mouse	Bone marrow, GM-CSF, 5 days	insulin; allopeptides	*in vivo*	Long-term survival of the graft; Immune tolerance restoration	Prevention of graft rejection	Thomas *et al.*, 2013 [176]; Yan *et al*, 2014 [177]
IL-10 and TGF-β	human	Blood monocytes, GM-CSF and IL-4, 5–6 days	insulin and GAD65; β2-glycoprotein I	*in vitro*; pre-clinical	Maturation-resistant phenotype, IL10/IL12; Reduced antigen specific T cell response	Antiphospholipid syndrome; Type 1 Diabetes	Segovia-Gamboa *et al.*, 2014 [178]; Torres-Aguilar *et al.*, 2012 [179]
Cholera toxin B	human	Blood monocytes, GM-CSF and IL-4, 5–6 days	-	*in vitro*	Maturation-resistant phenotype; Reduce T cell priming; regulatory T cell induction	Immune-mediated diseases	D’ambrosio *et al.*, 2008 [180]
Gene therapy, IL-10 plus TGF-β	rat	Bone marrow, GM-CSF, 5 days	-	*in vivo*	Long-term survival of the graft; Maturation-resistant phenotype	Prevention of graft rejection	Chen *et al.*, 2014 [181]
Gene therapy; silencing; IL-12/IL23/CD40/CD80/CD86/RelB	mouse	Bone marrow, GM-CSF or GM-CSF and IL-4, 5 days	collagen II; MOG petide; islet lysate	*in vivo*	Reduce disease severity and joint erosion; Reduce T cell priming; Reduced islet-specific T cell response; Reduce severity of Type 1 Diabetes	CIA (Rheumatoid arthitis); EAE; Type 1 Diabetes	Li *et al.*, 2012 [182]; Zheng *et al.*, 2010 [183]; Kalantari *et al.*, 2014 [184]; Ma *et al.*, 2003 [185]; Machen *et al.*, 2004 [186]

### 6.2. Pharmacologic Intervention

One of the most popular protocols to generate tolDCs is through pharmacologic blockage of the maturation process leading to a decreased susceptibility to be activated by PAMPs, DAMPS or pro-inflammatory cytokines [146,151,154,155,158,160,187]. Most used tolerogenic inducers are dexamethasone (Dex) and 1α,25-dihydroxyvitamin D3. Both drugs induce a semi-mature phenotype on DCs with intermediate expression of co-stimulatory molecules, such as MHC-II and CD86, being resistant to maturation stimuli and suppressing T cell activation [151,152]. Dex could also modulate the Nuclear Factor Kappa B (NF-κB) pathway, inflammatory cytokines, chemokines, and Ag-presenting molecules [148,150,188,189] (Figure 3B). In addition, it has been reported that Dex plus monophosphoryl lipid A stimulation on DCs induces a classical tolerogenic phenotype together with a high expression of CCR7 and CXCR4 chemokine receptors involved in leukocyte migration to lymphoid organs exhibiting a recruitment/migration response to CCL19 and CXCL12 [153] (Table 1). Remarkably, a Phase I clinical trial (AutoDECRA) based on the administration of autologous tolDCs generated by Dex and VD3 is being conducted in RA patients (currently recruiting patients) [ClinicalTrials.gov Identifier: NCT01352858] (Table 2). It is important to note that tolDCs will be administered arthroscopically into the involved joints. Results are not available yet. Although it has been reported that tolDCs generated by VD3 from MS patients induce hyporesponsiveness of myelin-speciﬁc T cells, clinical trials based on the therapeutic use of tolDCs MS are still ongoing [156,157,190,191].

Aspirin (Acetylsalicylic acid) inhibits CD40, CD80, CD86, and MHC class II expression on DCs, decreases NF-κB signaling and induces expression of immunoglobulin-like transcript 3 (ILT3), a T cell inhibitor, suggesting it to be an important factor in tolDC function [160,161]. Aspirin treated DCs showed an immature morphology and failed to stimulate T cells in mixed lymphocyte reaction [160]. In addition, in DCs, aspirin inhibits phagocytosis and modulates the expression of endosomal SNAREs (Soluble NSF attachment protein receptors), affecting the uptake of Ags for processing and presentation to T cells, thus preventing the immune response [162]. Niflumic acid is a non-steroidal anti-inflammatory agent which shows a potential tolerogenic effect on DCs decreasing the expression of CD80 while increasing the expressions of the co-inhibitory molecules ILT3 and ILT4 [192].

Rapamycin has been extensively reported to exhibit tolerogenic potential. mTOR inhibition by rapamycin (RAPA) promotes tolDCs that induce Treg expansion *in vivo* and *in vitro*, as well as inhibition of effector T cell proliferation [158,159]. RAPA binds to FKBP12 thus inhibiting mTOR, which exerts different cellular functions, including modulation of activation, proliferation and regulating cellular metabolism [158] (Figure 3B). Interestingly, when DCs were exposed to RAPA during differentiation they showed a global reduction in the expression of co-stimulatory molecules CD40, CD80 and CD86, as well as in inhibitory receptors, such as ILT2, ILT3 and ILT4, which is not consistent with a tolerogenic phenotype [159]. In contrast, another study has shown that DCs from transplanted patients treated with RAPA showed an increase in ILT3 and ILT4 expression [193]. RAPA suppresses IL-4-dependent maturation of DCs by down-regulation of IL-4 receptor complex (CD124, CD132). Moreover, RAPA prevents DCs expansion *in vivo* induced by Flt3-L, and impairs LPS-induced expression of the co-stimulatory molecules CD80 and CD86 while suppresses the production of TNF-α and IL-18 [158,187,194] (Table 1).

**Table 2:** Clinical studies that are in progress based on the experimental use of tolDCs for autoimmune diseases treatment. AutoDECRA: autologous tolerogenic dendritic cells for rheumatoid arthritis; IL: interleukin; GM-CSF: granulocyte monocyte colony stimulating factor.

Protocol for DC					Name	Targeted Disease	Results/Status	ClinicalTrials.gov Identifier
Agent	Origin	Differentiation	Type of Study	Route				
Dexamethasone and Vitamin D3	Blood monocytes	GM-CSF and IL-4, 5–6 days	Phase I; Proof of safety	Arthroscopically	AutoDECRA	Rheumatoid arthitis	No study results posted; Ongoing study	NCT01352858
BAY11-7082	Blood monocytes	GM-CSF and IL-4, 5–6 days	Phase I; Proof of safety	Intradermally	-	Rheumatoid arthitis (citrunillated peptides)	Safe and well tolerated; Ongoing study	-
Gene therapy; siRNA; CD40/CD80/CDD86	Blood monocytes	GM-CSF and IL-4, 5–6 days	Phase I; Proof of safety	Intradermally	-	Type 1 Diabetes	Safe and well tolerated; Ongoing study	NCT00445913
Low GM-CSF	Blood monocytes	low GM-CSF, 6 days	Phase I; feasibility study	Intravenous	The One Study	Kidney transplant	No study results posted; Ongoing study	-

Pharmacologic or genetic modulation of heme oxygenase-1 (HO-1) has been shown to exert different immunoregulatory properties, thus HO-1 is currently a therapeutic target for tolDCs generation. This enzyme catabolyzes heme degradation into Fe^2+^, biliverdin and carbon monoxide (CO). In addition, the administration of its metabolic product, carbon monoxide (CO) would also display different immunoregulatory properties. HO-1 expression can be induced by Cobalt Protoporphyrin (CoPP, a heme group analog), both *in vitro* and *in vivo*, preventing DCs maturation by LPS without affecting IL-10 production [163]. In addition, DCs exposed to CO by the administration of CO-releasing molecules show alterations in antigen presentation and a reduced capacity of T cell priming [195]. Interestingly, CO-treated autologous DCs reduce autoimmunity in a diabetic transgenic model by blocking β1-integrin expression in autoreactive CD8^+^ T cells, reducing their capacity to infiltrate the pancreas [196]. One of the most important cellular events on DCs after sensing a maturation stimulus is the nuclear translocation of NF-κB [197]. Pharmacologic interference with NF-κB signaling pathway has been successfully employed to generate tolDCs. DCs treatment with BAY11-7085 or andrographolide, two NF-κB inhibitors, induces Treg expansion and modulates experimental autoimmune arthritis and EAE respectively [133,149,164] (Figure 3B). In addition, NF-κB inhibition by BAY11-7085 on DCs prevents CD40 and HLA-DR expression, as well as cytokine production after NiSO stimulation without major changes in the expression of CD86 and CD83 [165]. Interestingly, a small study in RA patients based on the administration of BAY11-7082-generated tolDCs in RA has revealed promising results (Table 2). TolDCs were loaded with citrullinated autoantigen peptides (cit-vimentin 447–455, cit-fibrinogen beta chain 433–441, cit-fibrinogenalphachain 717–725, cit-collagen type II 1237–1249) in order to drive an Ag-specific tolerogenic response and given to patients intradermally. Two initial doses were assayed with increasing subsequential administrations, detecting only minor effects in patients. A significant improvement was achieved in the group of patients with most active disease while those with a low activity score mostly remained with a stable condition [198].

Tacrolimus could also promote the generation of tolDCs displaying an immature phenotype characterized by the induction of IL-10 and TGF-β mRNA while reducing the production of TNF-α and limiting the proliferation of effector T cells [166]. Remarkably, the administration of tacrolimus-generated tolDCs to arthritic mice ameliorated disease and progression mainly by altering Th1/Th17 profiles in the spleen [166].

In addition, other anti-inflammatory mediators, such as deoxyspergualin, mycophenolate mofetil, and spironolactone, also have been shown to induce a tolerogenic phenotype on DCs [133,199,200].

### 6.3. Biological Compounds

IL-10 is the most common biological agent that induce tolDCs via IL-10R/Jak/STAT signaling that regulating several anti-inflammatory genes [201]. In DCs, IL-10 signaling interferes with pro-inflammatory pathways, such as PI3K/Akt, NF-κB, TLR/IRAK/TRAF6/MyD88, MAPK and Ras/Raf conferring a tolerogenic phenotype [167,168,202]. IL-10 stimulation of DCs decreased the expression of both co-stimulatory molecules, CD80 and CD86, as well as the inhibitory molecule PD-L1 and the absence of CD80 or CD86 leads to a reduced capacity of suppress a delayed-type hypersensitivity response [174] (Figure 3C). IL-10 prevents DC maturation and increases mRNA expression of several inhibitory receptors such as ILT2, ILT3, ILT4, ILT5, DCIR, PILRA, FcγRIIb and SLAM which may improve the global tolerogenic function [169]. It has been reported that the administration of tolDCs generated by IL-10 prolongs allograft survival by blocking the expression of the co-stimulatory molecule CD86, leading to apoptosis of allospecific T cells [175]. IL-10-treated DCs induce antigen-specific T cell anergy, blocks proliferation and IL-2 and IFN-γ production [170,171,172,178,179]. Interestingly, an analog peptide to IL-10 was capable of inducing TGF-β production by human DCs [173].

Although it has been reported that DCs from lupus patients show an altered phenotype, IL-10 treatment successfully induced tolDCs with a decreased capacity of T cells priming [28]. Unfortunately, the use of tolDCs in clinical trials of lupus patients still remains to be performed [6,28,200,203,204].

TGF-β has also been used as a tolerogenic agent. Similarly as observed with IL-10 treatment, TGF-β reduced the expression of CD80 and CD86 by DCs, together with a decreased secretion of the pro-inflammatory cytokine IL-12 and a reduced capacity to prime T cells [176] (Figure 3C). In *in vivo* assays, tolDCs generated with TGF-β delayed corneal allograft rejection and increased the number of Treg expressing Foxp3 and CTLA-4 [177]. Also, the administration of TGF-β-induced tolDCs to grafted β-cells islets prolongs graft survival suggesting an acquired tolerogenic phenotype which may ameliorate the immune mediated disease [176]. Similarly, Growth differentiation factor-15 (GDF-15) which is a member of the TGF-β superfamily also induces a tolerogenic phenotype in human DCs suppressing maturation, decreasing the expression of co-stimulatory molecules CD83 and CD86, reducing IL-12 production and inhibiting T cell priming [205].

Interestingly, cholera toxin B subunit could also induce tolDCs, which produce high amounts of IL-10 and reduce the ability to stimulate T cells in a mixed lymphocyte reaction [180]. Also, it has been reported that cholera toxin B could suppresses TNF-α secretion by DCs and induced IL-10 production [206].

### 6.4. Gene Therapy

Alternative protocols to induce tolDCs such as gene therapy have been growing during the last years. The interference RNA (RNAi) technology and the new gene transfer systems offer multiple options to generate tolDCs by the transfection of short hairpin RNAs, microRNAs, small interfering RNAs and the use of viral or synthetic vectors containing tolerogenic genes [207,208,209] (Figure 3D). In the liver model of transplant rejection, tolDCs generated by the co-transfection of IL-10 and TGF-β improve liver graft survival and decrease serum IL-12 levels [181] (Figure 3D). IL-12 gene silencing by siRNA induced tolDCs and the transfer of these cells to collagen induced arthritis (CIA) mice ameliorates disease mainly by suppressing of T and B cells responses [182]. Similarly, gene silencing by siRNA transfection of the classical co-stimulatory molecules CD40, CD80, and CD86 also ameliorated disease in the CIA mice model of RA [183,210]. A gene therapy approach based in the transduction of monocytes with an adenovirus vector overexpressing HO-1 gene results in a reduction of nitric oxide and TNF-α release, augmenting IL-10 production after LPS stimulation [211]. Also, the administration of tolDCs generated by lentiviral vector transduction expressing shRNA specific for CD40 and IL-23 ameliorate clinical score in the EAE model and decreases IL-17 while increases IL-10 production [184]. Similarly as observed with pharmacological induction of tolDCs, interfering with NF-κB signaling has also been successfully performed by gene therapy with the lentiviral transduction expressing specific shRNA for RelB (NF-κB subunit) on DCs which in turn reduced maturation, decreased pro-inflammatory cytokines and co-stimulatory molecules expression [212]. Furthermore, it has been shown that the administration of oligodeoxynucleotides specific for NF-κB binding sites to DNA can inhibit NF-κB activity leading to a phenotype resistant to maturation in DCs [185]. In addition, the transfer of NF-κB-specific ODN tolDCs or CD40/CD80/CD86-antisense ODN tolDCs to NOD mice ameliorates clinical symptoms of Type 1 diabetes, induces islet-specific T cell hyporesponsiveness and increases the prevalence of regulatory T cells in the spleen [185,186] (Table 1). The administration of antisense (Figure 3D). These new experimental approaches promote the implementation of lentiviral technology targeting DC-T cell or DC-B cell interactions crucial for autoimmune diseases pathogenesis.

Remarkably, a Phase I Clinical trial in patients with type 1 diabetes was carried out to evaluate the safety of the injection of autologous tolDCs generated by the transfection of siRNA targeting co-stimulatory molecules CD40, CD80, and CD86 [213,214]. The procedure was well-tolerated and no severe adverse reactions were reported (ClinicalTrials.gov identifier NCT00445913) (Table 2) [213].

In the setting of inflammation DCs are crucial in maintaining immune surveillance of peripheral tissues and initiating the immune responses inside the draining lymph nodes [215]. Similarly, peripheral tolerance to tissue specific antigens could be achieved by the homeostatic presentation of autoantigens by immature DCs in the draining lymph node [216]. Different immune cell interactions could take place in lymph nodes to promote tolerance [217,218]. It has been demonstrated that in lymph nodes from NOD mice, Tregs prevent the interaction and cell arrest of effector T cells with DCs, reducing autoimmune responses [217]. Furthermore, disruption of PD-1–PD-L1 interactions in the lymph node can enhance the interactions of self-antigen loaded DCs with “tolerized” T cells leading to T cell priming and autoimmunity in NOD mice [219]. Alternatively, local injury could led to tissue destruction by infiltrating immune cells increasing the recruitment of DCs that migrate to the draining lymph node leading to disease amplification, epitope spreading, autoimmunity and organ failure [220]. In contrast, during systemic autoimmune diseases, such as lupus, target autoantigens, including histone, Ro/La and DNA, are widely expressed leading to systemic lymphoproliferation and massive lymphadenopathy [221]. Thus, while in organ-restricted autoimmunity most self-specific T cells stay in draining lymph nodes, during systemic autoimmunity pathogenic T and B cells are located at widespread lymph nodes resulting in a greater number of autoreactive cells than organ-restricted autoimmune diseases [220]. This feature of pathogenic cells during systemic autoimmunity has hindered the development of new therapies based on tolDCs mainly due to that the full effect could be achieved after suppressive cells migrate to distant lymph nodes as well as compromised tissues [200,222]. Based on this notion, tolDC based therapy in organ-restricted autoimmune diseases should be more easily achieved by directly administering tolDCs near to draining lymph nodes or compromised tissue [223] (Clinical Trials.gov Identifier: NCT01352858). It has been shown that after DCs are injected intravenously, they migrate to different organs including spleen, liver, lungs and lymph nodes [224,225]. The migratory capacity of tolDCs to lymph nodes is essential for immunesuppression and is thought to be dependent on CCR7 expression [153,226]. These data highlight the importance of choosing tolerogenic agents that induce the expression of chemokines receptors that allow the entry of tolDCs into lymph nodes.

Although several self-Ags have been described in SLE, such as nucleosomes, Ro, La and Sm, the identification of the most immunodominant T cell self-antigens in lupus or systemic autoimmunity are still lacking [200,227]. Understanding the pathogenesis of self-antigens is crucial for developing a novel therapy based on specific immunesuppression by tolDCs. Meanwhile, the use of self-antigen loaded tolDCs with nucleoproteins would be a potential therapy for lupus treatment which may restore immune tolerance to specific antigens [200]. Achieving this goal would avoid systemic immunesuppression without impairing the immune system and preventing the increased susceptibility to opportunistic infections secondary to pharmacologic therapy [3].

To transfer the DCs-based technology to clinical practice, standardization of generation and assessment protocols is mandatory to ensure validity of the clinical trials and quality of the therapeutic approach. Researchers must first define the precursors from which DCs will be generated. Since monocytes can be easily obtained these are the most common source for DC generation, but others cell types such as CD34^+^ progenitors from human cord blood may be used as well [228]. The isolation method must provide high purity of DC-precursors and be appropriate for clinical applications, such as immunomagnetic separation or elutriation. In addition, cell culture variables including duration of the differentiation step, medium composition and density of cultured DCs-precursors must be adjusted to improve yield and viability. Tolerogenic phenotype must be evaluated by the measurement of co-stimulatory molecules expression, chemokine receptors, secretion of pro- or anti-inflammatory cytokines. Assays to evaluate tolerogenic function of tolDCs must be adapted to the current knowledge of the disease to be targeted by the therapy. For example, tolDCs designed for RA are commonly pulsed with synovial fluid or a mixture of citrullinated peptides to evaluate antigen-specific T cell suppression. However, mixed lymphocyte reaction assays may be a suitable approach to evaluate the tolerogenic capacity of tolDCs for most autoimmune diseases [198,200]. Cryopreservation is a crucial procedure to preserve viability and function of tolDCs when transferred from the manufacturating facility to the clinic [229,230]. Since thawing cells may result in cell death, viability assays should be performed after defrosting [231]. Additionally, cryopreservation itself could affect cell function without striking viability detriment and functional assays should be carried with thawed cells to evaluate if therapeutic potential is preserved [232]. Controlled freezing rate and slow thawing may be needed to optimize cryopreservation and to obtain fully functional tolDCs. Once tolDCs are fully characterized and optimal preservation conditions are defined, manufacture process should be translated to an adequate facility and acquire cell culture procedures to achieve GMP compliance to assure the quality and success of the cellular therapy. This implies adopting strategies to control collection, processing, storage and delivery of the product with high standard of quality [233].

## 7. Conclusions

Although tolDCs have been successfully generated by different methods, such as pharmacological or biological agent intervention and gene therapy, the precise role of DC-T cell and DC-B cell interactions in the global tolerance capacity still remains to be elucidated. Dex, VD3 and NF-κB inhibitors are the most used compounds to generate tolDCs. Understanding immunodominant self-Ags driving autoimmune responses are crucial in designing specific tolerogenic responses in order to make more efficient the tolDCs approaches and thus avoiding systemic immunosuppression. Due to the complexity of systemic autoimmunity, performing therapies on SLE patients, based on tolDCs, may be more difficult than approaches in tissue specific autoimmune diseases. Remarkably, clinical trials based on the generation of autologous tolDCs and subsequent transfers to autoimmune disease patients are already being conducted. Although much research needs to be performed, the success of the tolDCs approach may have a major clinical impact being a worthy challenge.

## Abbreviations

APCs: Antigen presenting cells
ANA: Anti-nuclear antibodies
BAFF: B cell activating factor
BLyS: B lymphocyte stimulator
cDCs: Conventional dendritic cells
CIA: Collagen induced arthritis
CoPP: Cobalt Protoporphyrin
DAMPs: Danger-associated molecular patterns
DCs: Dendritic cells
Dex: Dexamethasone
EAE: Experimental Autoimmune Encephalitis
HO-1: Hemeoxygenase 1
IC: Immune complex
ILT: Immunoglobulin-like Transcript
MS: Multiple sclerosis
mTOR: Mammalian target of rapamycin
NF-κB: Nuclear factor kappa B
PAMPs: Pathogen-associated molecular patterns
PD-1: Programmed death 1
pDCs: Plasmacytoid dendritic cells
PPAR: Peroxysome proliferator-activated receptor
RA: Rheumatoid Arthritis
RAPA: Rapamycin
RNAi: interference RNA
SLE: Systemic Lupus Erythematosus
SS: Sjögren’
s syndrome; T1D: Type 1 Diabetes
Th: T helper
TLRs: Toll Like Receptors
Treg: Regulatory T cells
VD3: 1α,25-dihydroxyvitamin D3
